# Peptide nanosponges designed for rapid uptake by leukocytes and neural stem cells†

**DOI:** 10.1039/c8ra00717a

**Published:** 2018-04-30

**Authors:** Asanka S. Yapa, Hongwang Wang, Sebastian O. Wendel, Tej. B. Shrestha, Nilusha L. Kariyawasam, Madumali Kalubowilage, Ayomi S. Perera, Marla Pyle, Matthew T. Basel, Aruni P. Malalasekera, Harshi Manawadu, Jing Yu, Yubisela Toledo, Raquel Ortega, Prem S. Thapa, Paul E. Smith, Deryl L. Troyer, Stefan H. Bossmann

**Affiliations:** Department of Chemistry, Kansas State University Manhattan KS 66506 USA sbossman@ksu.edu +1-785-532-6666 +1-785-532-6817; Department of Anatomy & Physiology, Kansas State University Manhattan KS USA troyer@vet.k-state.edu +1-785-532-6405; Microscopy and Analytical Imaging Laboratory, University of Kansas Lawrence KS USA

## Abstract

The structure of novel binary nanosponges consisting of (cholesterol-(K/D)_*n*_DEVDGC)_3_-trimaleimide units possessing a trigonal maleimide linker, to which either lysine (K)_20_ or aspartic acid (D)_20_ are tethered, has been elucidated by means of TEM. A high degree of agreement between these findings and structure predictions through explicit solvent and then coarse-grained molecular dynamics (MD) simulations has been found. Based on the nanosponges' structure and dynamics, caspase-6 mediated release of the model drug 5(6)-carboxyfluorescein has been demonstrated. Furthermore, the binary (DK20) nanosponges have been found to be virtually non-toxic in cultures of neural progenitor cells. It is of a special importance for the future development of cell-based therapies that DK20 nanosponges were taken up efficiently by leucocytes (WBC) in peripheral blood within 3 h of exposure. The percentage of live cells among the WBC was not significantly decreased by the DK20 nanosponges. In contrast to stem cell or leucocyte cell cultures, which have to be matched to the patient, autologous cells are optimal for cell-mediated therapy. Therefore, the nanosponges hold great promise for effective cell-based tumor targeting.

## Abstract 2

Nanosponges for drug delivery.

## Graphical abstract

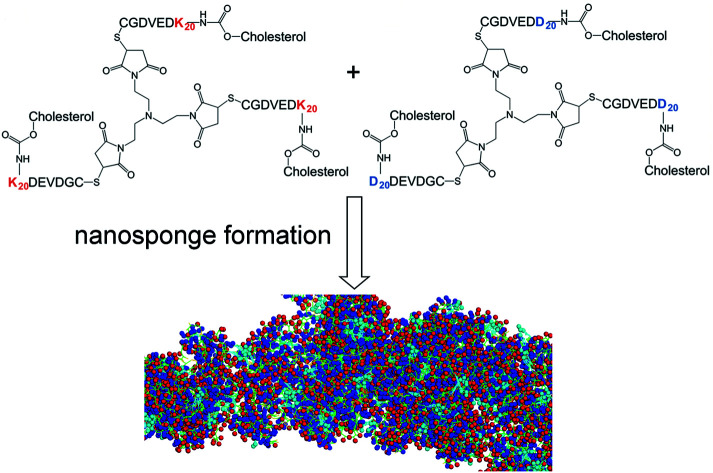

## Introduction

**Scheme 1:**
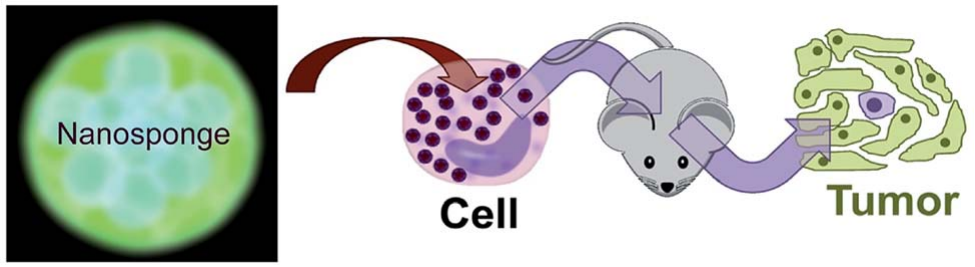
Principles of cell therapy utilizing nanosponges. Neutrophils in peripheral blood will be loaded by targeting them with peptide nanosponges. After the blood has been given back intravenously to the patient, it is anticipated that the neutrophils will home to tumors within 6–12 h. Alternatively, neural stem cells can be cultured, loaded with peptide nanosponges and injected intravenously into the patient.

One of the grand challenges in nanomedicine is the effective targeting of tumors and metastases.^1^ For almost a generation, Enhanced Permeation and Retention (EPR),^2,3^ the passive diffusion of nanosize delivery vehicles (*e.g.* vesicles,^4^ liposomes,^5^ exosomes,^6^ nanoparticles,^7^ polymer-based nanostructures^8–10^) through gaps in the vasculature that have been built rapidly around tumor tissue, has been hailed as an important breakthrough in the fight against cancer. Unfortunately, emerging evidence clearly suggests that the EPR effect works well in rodent models (especially in nude mice), but not in humans, who feature distinctly different vasculature and, compared to rodents, significantly slower tumor growth.^2,3,11^ Therefore, alternative targeting approaches are urgently needed. Active targeting strategies use either antibodies,^12–14^ antibody-fragments,^15–17^ peptide sequences^15,16,18^ or aptamers,^15,16^ which are capable of targeting receptors that are overexpressed in solid tumors, as for instance members of the integrin family.^19,20^ However, active targeting processes can be impaired by physiological barriers, such as high interstitial fluid pressures and the formidable physical barrier imposed by tumor stroma.^21^ Therefore, cell-mediated transport of anticancer drugs into the tumor tissue is, in the opinion of the authors, the most viable strategy to develop intelligent alternatives to chemotherapy.^10,22–27^ Transport cells have the ability to migrate to tumors and metastases following cytokine/chemokine gradients.^28^ Among them are stem cells,^29^ monocytes/macrophages^30,31^ and neutrophils.^32,33^ Neural stem cells, which can be, principally, cultured and matched to patient-types, have been successfully utilized for cell-mediated therapies in rodent models,^22,27,34^ as well as neutrophils^35,36^ and monocytes.^10,23,24,27^ The use of autologous cells has the potential of developing truly patient specific therapies, and also of significantly lowering the regulatory barriers for cell-based human cancer therapies.^37^ Targeting neutrophils and monocytes in peripheral blood will avoid the necessity for their time-consuming isolation and culturing, and further reduce the regulatory hurdles since cell isolation is not necessary. In Scheme 1, the principles of cell-based cancer therapy are shown. In step 1, the selected transport cell type is targeted. In order to maintain high transport cell viabilities, carrier particle (vector) recognition and uptake by the transport cell(s) has to be very efficient. Furthermore, the vector that is used to facilitate uptake has to be virtually non-toxic. After the cells have been returned to the host, they actively migrate to tumors and metastases following cytokine/chemokine gradients. The last step consists in the triggered release of the payload and uptake of the latter by the tumor and stromal cells.^27^

**Fig. 1:**
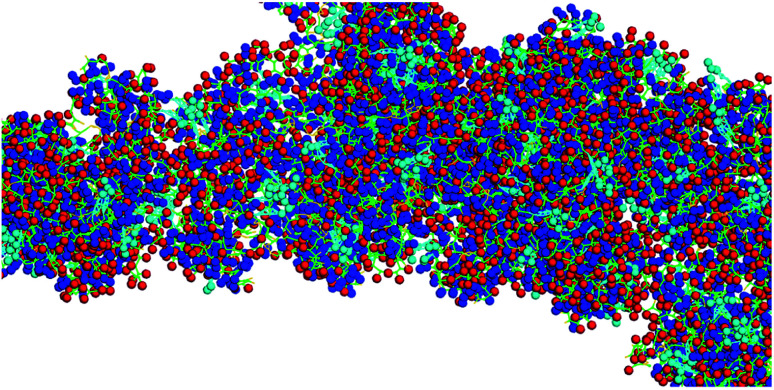
Typical structure of a nanosponge according to coarse grained molecular dynamics simulations, which are described in detail in ref. 38. Red: aspartate groups, blue: lysine groups, cyan: cholesterol aggregates, green: peptide backbone.

This team has recently reported the design, synthesis, and characterization of designer peptide-nanosponges for efficient uptake by delivery cells in drug delivery.^38^ Their supramolecular building blocks consist of (cholesterol-(K/D)_*n*_DEVDGC)_3_-trimaleimide units featuring a trigonal maleimide linker to which either lysine (K)_20_ or aspartic acid (D)_20_ are attached. Furthermore, a consensus sequence for caspase-6 (DEVD-GC)^39^ is integrated into the structures. This consensus sequence can also be activated by other executioner caspases, such as caspase-3, and -7. There is emerging evidence that caspases can actively contribute to the development and progression of tumors.^40^ This is in agreement with clinical evidence for the presence of active caspases in tumors.^40^ Caspases −3 and −6 are taken up by cells and are, therefore, suitable to cleave the consensus sequences of nanosponges that have been taken up by transport cells, thus triggering their release by means of apoptotic processes, which enhance the porosity of the transport cells and then dissect them into apoptotic bodies.^41^ Both, (cholesterol-(K)_20_DEVDGC)_3_-trimaleimide and mixtures of (cholesterol-(K)_20_DEVDGC)_3_-trimaleimide and (cholesterol-(D)_20_DEVDGC)_3_-trimaleimides form stable nanosponges (short notation: DK20). The structure of the novel nanosponges was investigated through explicit solvent and then coarse-grained molecular dynamics (MD) simulations. As Fig. 1 indicates, the nanosponge structure is featuring aspartate- and lysine-rich regions, together with cholesterol domains and (aqueous) solvent filled nanoholes. The resulting structure is fluctuating, depending on the temperature. Upon mixing with aqueous buffers DK20, nanosponges are immediately formed. They possess very low polydispersities and are long-term stable (up to 72 h as experimentally determined). They are capable of incorporating fluorescent dyes (*e.g.* carboxyfluorescein (discussed here) or PKH26 (ref. 38)), which can be used for fluorescence imaging and payload release studies.

It is noteworthy that the Coarse Grained MD Simulations did not result in spherical nanosponges due to the limited number of small trigonal units in these simulations. However, as shown below in Fig. 3, the principal findings from these simulations were corroborated by means of TEM.

**Fig. 2:**
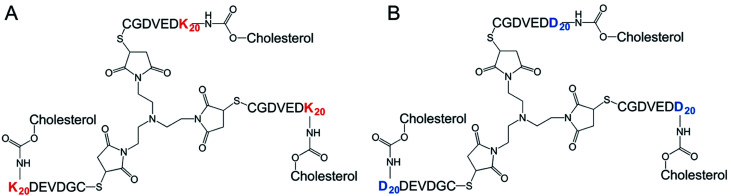
Tri-maleimide based peptide structures: components for the spontaneous formation of type DK20 nanosponges. (A) Lysine-based component K20 (MW = 11 334.74 g mol^−1^); (B) aspartic acid-based component D (MW = 10 439.51 mol^−1^).

**Fig. 3:**
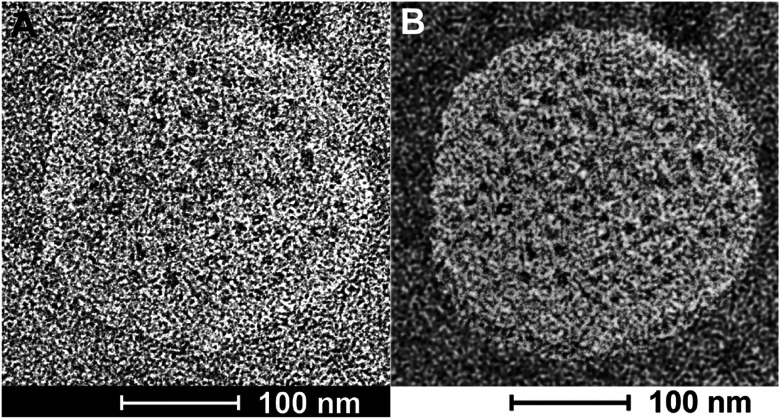
(A) TEM image of type DK20 nanosponges on HOPG, as deposited from PBS solution. Bright field transmission TEM (200 kV) of a type DK20 nanosponge. Water-filled vesicles are discernible as dark spots within the bright nanosponge. (B) Same image as in 3A after black/white correction filter function in Photoshop.

In order to function properly, the nanosponges should be taken up quickly by the transport cells, transported to the tumor sites, and then released. The latter will be achieved by means of programmed cell death (apoptosis), which will occur naturally in neutrophils 12 to 24 h after reaching the tumor environment,^42^ or by means of triggered apoptosis (other leucocytes and neural stem cells^27^). Caspase activation is the hallmark of apoptosis.^43^ We will utilize caspases, which are proteolytic enzymes, to activate the nanosponges for drug delivery purposes.

In this report, we will describe refined structural investigations by TEM, *in vitro* release studies of the model drug carboxyfluorescein by caspase-6 activation, as well as cell targeting experiments of cultured neural stem cells and leucocytes in peripheral (bovine) blood. The data obtained from these experiments will demonstrate the unique properties of type DK20 nanosponges.

## Experimental

### Synthesis and characterization of the nanosponges

The synthesis of all building blocks required for the assembly of DK20 and K20 nanosponges, as well as their characterization by NMR and MALDI-TOF has been described in an earlier report (Fig. 2).^38^

### TEM characterization

Samples for transmission electron microscopy (TEM) were prepared by dropping 10 μL of 0.050 mM type DK20 solution in PBS directly on a glow discharged TEM grid. Uranyl acetate was used as a positive staining agent in all TEM experiments. In all cases electron microscopy was performed at an accelerating voltage of 200 kV. Nanosponge morphology on HOPG was examined by bright-field and dark-field transmission electron microscopy (TEM) using a FEI Technai G_2_ transmission electron microscope at an electron acceleration voltage of 200 kV. Dark-field TEM did not reveal a characteristic diffraction pattern. High resolution images were captured using a standardized, normative electron dose and a constant defocus value from the carbon-coated surfaces. All TEM measurements were performed at the Microscopy and Analytical Imaging Laboratory of the University of Kansas.^44^

### Nanosponge formation and DLS characterization

The hydrodynamic diameter and polydispersity index (PDI) of the formed nanosponges were measured by dynamic light scattering (DLS, ZetaPALS, Brookhaven Instruments Corp., Holtsville, NY). All measurements were carried out at 25 °C, with 658 nm laser wavelength, and 90 degree detection angle. Data were collected from an average of three measurements over 60 seconds.

### Carboxyfluorescein encapsulation

Equal molar ratios of (cholesterol-(K)_20_DEVDGC)_3_-trimaleimide and (cholesterol-(D)_20_DEVDGC)_3_-trimaleimide (5.0 × 10^−4^ M of each component) were dissolved in 10 μM carboxyfluorescein PBS (pH = 7.4) solution. After incubating at room temperature for 2 hours, the solution was transferred to a 3500 Da molecular weight cutoff dialysis bag. Free carboxy-fluorescein was removed by means of continuous dialysis against 1 × PBS buffer until virtually no fluorescence could be detected in the solution using a Fluoromax-2 spectrometer. Using a fluorescence calibration curve, it was estimated that the concentration of free carboxyfluorescein was < 1 nM. At this point, a dark red color was still retained inside the dialysis bag. This finding provided a good indication that carboxyfluorescein had been trapped inside the peptide nanosponges. From the integrated UV/Vis-absorption of the dialysis solution we have estimated that 65 ± 4 mol% of carboxyfluorescein was encapsulated in the procedure. After lyophilizing to dryness, a yellow/brown powder was obtained, which could be easily re-dispersed in PBS by vortexing for 5 min. In a subsequent dialysis experiment, it was found that virtually no carboxyfluorescein was leached after 24, 48, and 72 h. The UV/Vis and fluorescence spectra of carboxyfluorescein, as well as the fluorescence calibration curve as a function of carboxyfluorescein concentration can be found in the ESI section (Fig. S1, S2, and S3†). The average number of encapsulated carboxyfluorescein molecules per DK20 nanosponge was estimated to 8.5 ± 2.

### Caspase-6 triggered dye release

The dye release experiment was performed using a fluorescence plate reader (BioTek Synergy H1). 200 μL of carboxyfluorescein loaded nanosponges in PBS solution (0.20 mg mL^−1^) were added to each well of a 96-well black clear-bottom plate. To each control well, 10 μL of PBS buffer was added, and to each experimental well, 10 μL of caspase-6 PBS solution (0.1 μg mL^−1^, 2.2 × 10^−7^ M, Enzo LifeSciences) was added.

The plate was incubated at 37 °C, the fluorescence intensity at 520 nm was recorded every 5 min.

### Cell experiments and MTT assays

The cytotoxicity of the PKH26 containing nanosponges was assessed by utilizing the MTT assay^45^ on C17.2 neural progenitor cells (NPCs),^34^ which were a gift from Dr. V. Ourednik (Iowa State University) to Dr. D. L. Troyer, DVM (Kansas State University, Anatomy & Physiology). NPCs were originally developed by Dr. Evan Snyder.^46^ These cells were maintained in DMEM supplemented with 10% FBS (Sigma-Aldrich), 5% horse serum (Invitrogen), 1% glutamine (Invitrogen), and 1% penicillin/streptomycin (Invitrogen). PKH26 is a hydrophobically modified cyanine 3.0 dye. The preparation of PHK26-loaded type DK20 nanosponges was described earlier.^38^ Cell experiments were carried out in the culturing medium described above. The percentage of viable cells was determined after 24 and 48 hours of incubation. Cells were seeded in T-25 flask. After 24 h of incubation at 37 °C, cells were re-plated in a 96 well plate at 20 000 cm^−2^ density and further incubated for 24 h at 37 °C to obtain 80% confluency before the nanosponges were added.

Concentration series of type DK20 nanosponges (0.0, 0.1, 0.2, 0.5, 1.0, 2.0, 5.0, 10, 20, 40, 60, 80, 100 μmol L^−1^ in total, molar ratio 1 : 1) were prepared by dissolving the nanosponge components in the same media that were used for culturing the cells. Cells were incubated for 24/48 h at 37 °C. Eight replicates were prepared for each concentration. A portion of 10 μL of MTT reagent (5 mg mL^−1^ in PBS) was added to each well, and the plates were incubated for another 4 h at 37 °C. Finally, 100 μL of 10% sodium dodecyl sulfate in 0.010 M HCl was added into each well and incubated for 24 h at 37 °C. Their absorbance was recorded by using a plate reader at 550 nm and 690 nm. PBS solution was used as control for all the experiments.

Murine stem cells were imaged by using a Zeiss, Axiovert 40 CFL microscope with darkfield, brightfield, phase contrast and epifluorescence illumination, a camera system and Jenoptik, ProgRes C3 Cool camera and a ProgRes Capture Pro 2.10.0.0 software.

### Cell uptake from peripheral blood

Bovine blood was obtained at the Kansas state feed lot. Blood was collected in citrated (0.105 M) 4.5 ml tubes (BD Vacutainer, Franklin Lakes, NJ, USA). The collected blood was pooled and split into 3.0 ml samples. Samples were supplemented with 1.0 mL of serum free RPMI medium to ensure supply of nutrients. The samples were incubated with 1.0 mL of 1.0 mg mL^−1^ type DK20 nanosponges in PBS at 37 °C. Leukocytes (WBC) were extracted *via* removal of the buffy coat after centrifugation.^47,48^ Red Blood Cell Lysis Buffer (Sigma-Aldrich, St. Louis, MO, USA) was used to remove any remaining red blood cells and the samples were washed with PBS (10 min, 500 g). Cells were counted *via* hemocytometer and diluted to achieve a concentration of 5 × 10^5^ cells per mL, suitable for analysis by flow cytometry (Guava EasyCyte, EMD Millipore). The survival of WBC incubated type DK20 nanosponges was detected with a annexin V/propidium iodine apoptosis kit (Novus Biologicals). The protocol provided with the kit was exactly followed.

## Results

### TEM-analysis of the nanosponge structure

Bright field transmission electron microscopy was able to reveal structures that are formed after depositing the nanosponges directly onto HOPG grids and exposing them to the high vacuum inside the TEM. The flattened nanosponges contain dark spots, which are indicatives of water/buffer-filled pockets inside the structure. Furthermore, after applying a black/white correction filter function available in Adobe Photoshop, brighter than average spots can be discerned within the nanosponge structure, which are indicative of cholesterol-rich regions. The average grey within the structure shown on Fig. 3B suggests the presence of both, lysine and aspartate-rich regions, which retain some of their water-content in high vacuum. These findings are in excellent agreement with the principal results of the molecular dynamics (MD) simulations of nanosponge structure.

It is noteworthy that the nanosponges, obtained under the experimental conditions described here, appear to be larger (240 ± 30 nm in diameter), whereas their diameter reported earlier was 90 ± 15 nm.^38^ However, a DK20 concentration approx. Five times lower (than reported in ref. 38).

### Caspase-6 triggered carboxyfluorescein release

**Fig. 4:**
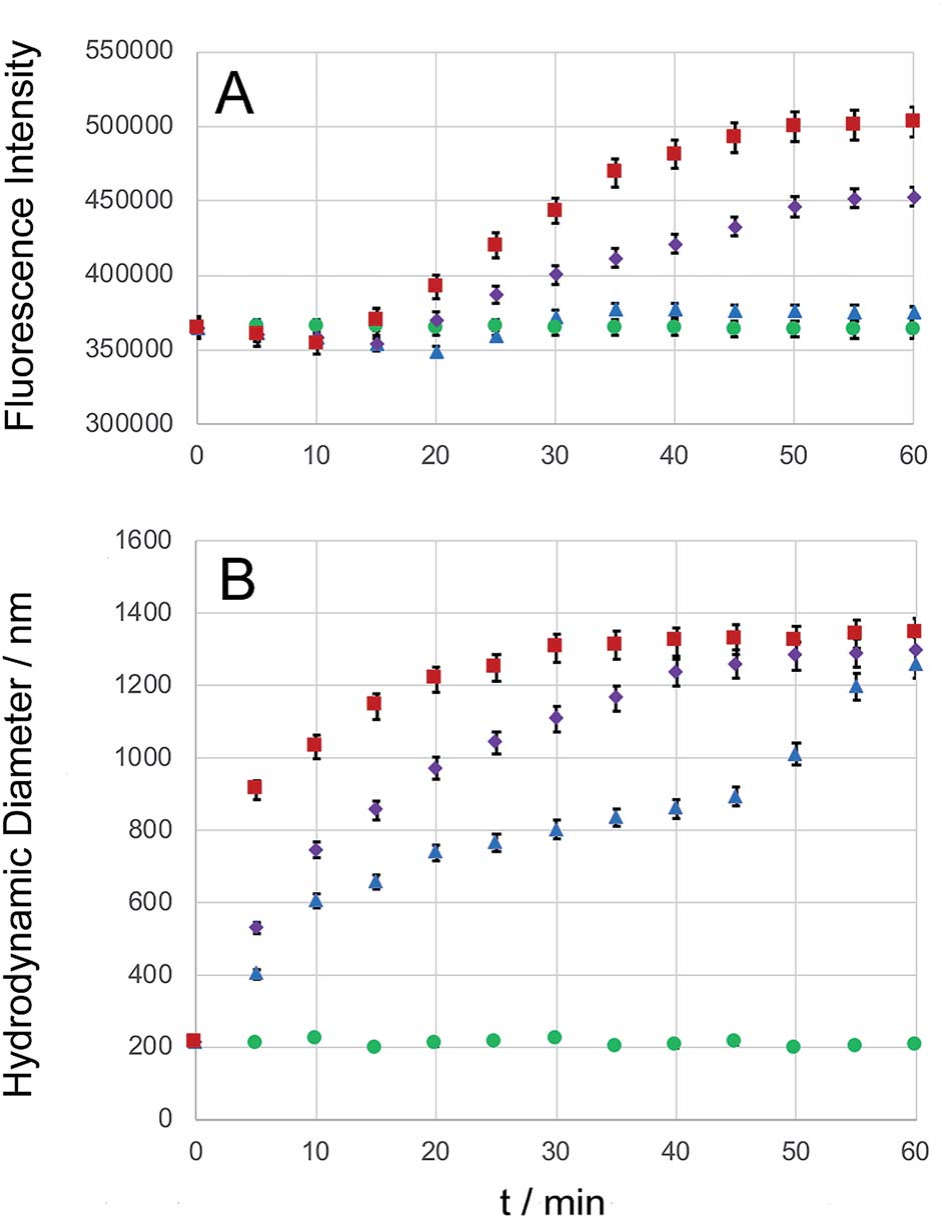
Caspase-6 triggered carboxyfluorescein (CF) release. Type DK20 nanosponges containing carboxyfluorescein (0.0005 M of D20 and K20, see ESI†) were incubated with PBS (control, green dots), as well as 1.0 × 10^−8^ M caspase-6 in PBS (blue triangles), 1.0 × 10^−7^ M caspase-6 in PBS (purple diamonds), and 1.0 × 10^−6^ M caspase-6 in PBS (red squares) at 37 °C (pH = 7.4). (A) The observed fluorescence emission intensity (relative units) occurring from carboxyfluorescein was recorded at 513 nm with a 5 nm bandpass filter, *λ*_exc_ = 493 nm, as a function of time. (B) Hydrodynamic diameters (measured by means of DLS) *vs.* time. Experimental errors are shown for all cases where they extend beyond the size of the symbols.

**Fig. 5:**
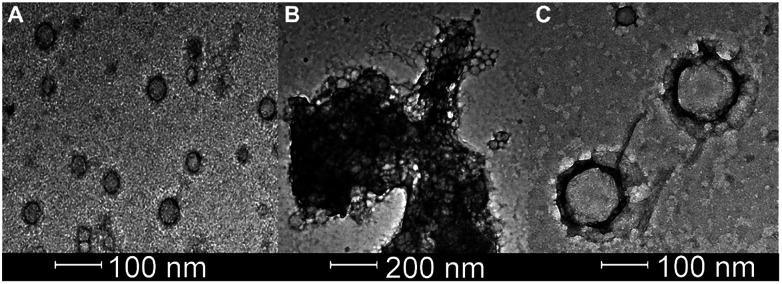
Bright field transmission TEM (200 kV) of 0.20 mg mL^−1^ of carboxy-fluorescein-loaded CF-DK20 nanosponges: (A) Nanosponges deposited from PBS before adding caspase-6. (B) Reactive mixture deposited from PBS containing caspase-6 (2.60 × 10^−10^ M) after 15 min of reaction at 37 °C. (C) Novel nanostructures, which were formed in the reaction, deposited from PBS containing caspase-6 (2.60 × 10^−10^ M) after 60 min of reaction at 37 °C.

Carboxyfluorescein is a fluorescent dye, which has been used for microscopy and cell-tracking purposes. It is established that at increased concentrations, carboxyfluorescein undergoes intense self-quenching.^9,49^ A detailed investigation of the concentration-dependent quenching of carboxyfluorescein in liposomes revealed both, monomer–monomer and monomer–dimer energy transfer processes.^50^ Carboxyfluorescein dimers are non-fluorescent. Because carboxyfluorescein fluorescence can increase as a function of decreasing dye concentration, it has become a popular probe detecting drug release from a delivery system.^9,51,52^ After entrapping carboxyfluorescein into type DK20 peptide nanosponges and subsequent lyophilizing to a powder, the obtained solid was dissolved in 3.0 mL PBS buffer (pH = 7.4). DLS measurements showed that the hydrodynamic diameter was 213 ± 25 nm before adding caspase-6 (see Fig. S4†). The nanosponges remained stable in PBS over a 24 h period. Dye release experiments using three concentrations of capase-6 are shown in Fig. 4A, together with the hydrodynamic diameters of the nanosponges, observed by means of DLS (Fig. 4B). The hydrodynamic diameters of the nanosponges steadily increased in the presence of caspase 6, indicating cleavage of the caspase-6 cleavage site DEVDGC,^39^ and subsequent formation of new micro-sized structures. At all three investigated concentrations of caspase-6, a maximum hydrodynamic diameter of 1300 ± 50 nm was detected my means of DLS, albeit at different reaction times. The observed fluorescence intensities of carboxyfluorescein were clearly a function of the added caspase-6 concentration. When 1.0 × 10^−8^ M of caspase-6 was added, the fluorescence intensity decreased during the first 25 min., which was followed by an increase. After 35 min. A plateau was reached, which corresponded to an overall fluorescence increase of 3.7 ± 0.1%. When 1.0 × 10^−7^ M of caspase-6 was added, the observed decrease in fluorescence was smaller, and a plateau corresponding to an overall fluorescence increase of 24.4 ± 0.5% was reached after 55 min. When 1.0 × 10^−6^ M of caspase-6 was added, the observed decrease in fluorescence was smallest, followed by the largest observed increase in fluorescence intensity (38.2 ± 0.9% after 55 min.). The observed concentration-dependent decrease/increase pattern suggest a rearrangement of nanosponge structure during caspase-6 digestion. This hypothesis is corroborated by TEM results, which are shown in Fig. 5. As shown in Fig. 4A, the amount of released carboxyfluorescein is clearly dependent on the concentration of caspase-6 that was added. The use of PBS medium ensured that the pH of all systems was 7.4. However, due to the observed rearrangement of the nanosponge structure after enzymatic cleavage, the amount of released fluorescent dye is not a linear function of the observed fluorescent increase. Nevertheless, the experimental data summarized in Fig. 4 clearly demonstrates the potential of DK20 nanosponges for cell-based drug delivery. It should be noted that DK20 nanosponges in PBS containing 5 percent (by volume) of human serum were stable for 24 h, indicating virtually no proteolytic cleavage. This result is important, because it demonstrated that DK20 nanosponges are at least short-term stable in the presence of serum, due to the absence of active caspases. This is of importance for the uptake experiments of type DK20 nanosponges by leucocytes in peripheral blood discussed below. At higher concentrations of serum in PBS, DLS could not be utilized as a detection method due to the presence of thousands of human proteins.

### TEM-analysis of caspase-6 activation

Bright field TEM was also successfully used to visualize the effect of caspase-6 activation of carboxyfluorescein-loaded DK20 nanosponges. In Fig. 5, a sequence of three TEM images is shown: (A) 0.20 mg mL^−1^ of CF-DK20 nanosponges, deposited from PBS dispersion onto HOPG. (B) 0.20 mg mL^−1^ of CF-DK20 nanosponges after 15 min. Of incubation at 37 °C with commercially available caspase-6 (2.60 × 10^−10^ M), deposited from PBS dispersion onto HOPG. (C) 0.20 mg mL^−1^ of CF-DK20 nanosponges after 60 min. Of incubation at 37 °C with commercially available caspase-6 (2.60 × 10^−10^ M), deposited from PBS dispersion onto HOPG. TEM images were recorded immediately after the deposition of the (reactive) nanosponges on the carbon surfaces. Uranyl staining was added shortly before depositing the dispersions onto HOPG.

**Fig. 6:**
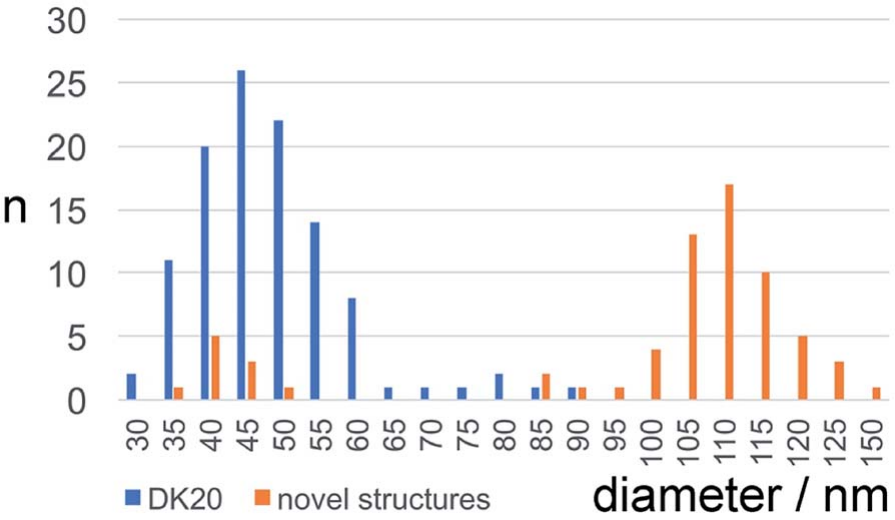
Size distribution of carboxyfluorescein-loaded DK20 nanosponges before and after “digestion” with caspase-6 (as measured by means of TEM, *n*: number of particles counted).

Fig. 5 shows that caspase-digestion of CF-DK20 nanosponges leads to the formation of a novel supramolecular structure. We have observed that the presence of a charged molecule (here: 5(6)-carboxyfluorescein) within the DK20 framework will influence the size of the formed aggregates. This effect is responsible for the differences in diameter that are observed compared to the TEM shown in Fig. 3 and ref. 38. Furthermore, the spherical nanosponges are deposited onto a carbon surface for the purpose of TEM. This will flatten their structures to a 2D coating and, at least partially, lead to the orientation of the hydrophobic cholesterol labels towards the carbon surface. Therefore, in contrast to dynamic light scattering, the nanosponges' structure will be somewhat distorted by the procedures necessary to record TEM. The resulting nanosponge diameters were 45 ± 10 nm (Fig. 6).

In Fig. 5B the originally observed organic structures have completely vanished and a mesh of organic structures has formed. It is our interpretation of this observation that caspase-6 was able to cleave at least a fraction of the DEVDGC, thus disrupting the structure of the nanosponges. Enzymatic cleavage releases cholesterol-K_20_-DE and cholesterol-D_20_-DE units, and according to the results shown in Fig. 5c, these units (or at least cholesterol-K_20_-DE) are able to form novel supramolecular structures. The re-formation of well-ordered structures may be responsible for the observed release of “only” about 3.7 ± 0.1% of carboxyfluorescein, which was observed by means of quantitative fluorescence recording. Interestingly, the supramolecular structures formed after 1 h of “digestion” with caspase-6 (2.60 × 10^−10^ M) are larger than the original nanosponges (110 ± 20 nm, Fig. 6).

### Cell toxicity of the peptide nanosponges

**Fig. 7:**
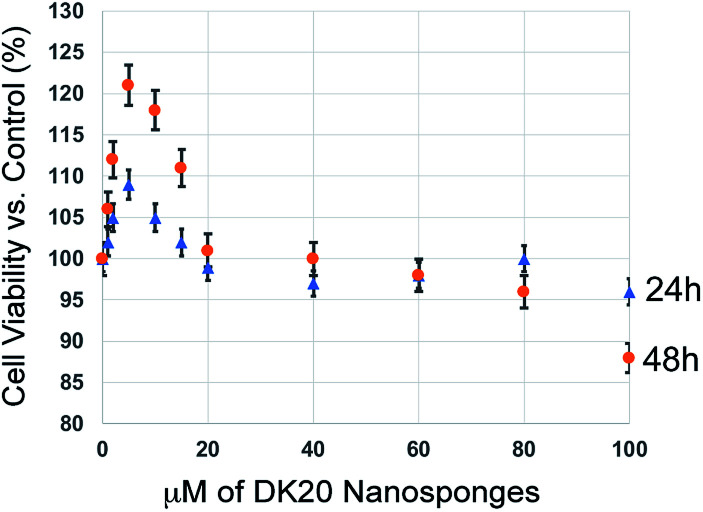
Cell viability of C17.2 neural progenitor cells (NPCs) as a function of DK20 nanosponge concentration and incubation time (24 h and 48 h), as measured by the MTT assay.^45^ Nanosponges were added to the cell culture medium in their respective concentrations (see ESI†). The cell viabilities after 24 h and 48 h in the absence of DK20 nanosponge, shown at 0 μM of DK20 nanosponges, were used as references to calculate viabilities.

We have performed classic MTT cell proliferation assays^45^ to determine the cell viability of murine C17.2 neural progenitor cells (NPCs)^34^ after incubation with DK20 nanosponges. DK20 nanosponges are not toxic to NPCs, even at concentrations as large as 100 μM (Fig. 7). Only a slight increase in cell proliferation was observed at lower concentrations.

### Nanosponge-uptake by leucocytes in peripheral blood

**Fig. 8:**
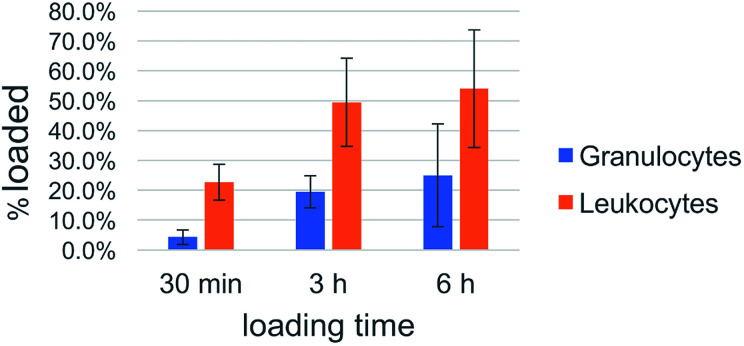
Uptake efficacy of type DK20 nanosponges by non-granulocyte leukocytes (mainly lymphocytes and monocytes) and granulocytes (neutrophils) in peripheral blood as function of incubation time.

Cell uptake kinetics were recorded to determine the uptake efficiencies of the peptide nanosponges by neutrophils and leukocytes in peripheral blood. The results are summarized in Table 1 and Fig. 8. They indicate that the targeting of defensive cells within peripheral blood, followed by cell-based transport to the tumor site, is a feasible treatment strategy.

**Table 1:** Uptake of DK20 nanosponges by leukocytes in peripheral blood

	DK20 Nanosponges					
	30 min		3 h		6 h	
	Neutrophils loaded	Other leukocytes loaded	Neutrophils loaded	Other leukocytes loaded	Neutrophils loaded	Other leukocytes loaded
Ave-rage	4.3%	22.6%	19.5%	49.4%	25.0%	54.0%
StDev	2.4%	6.0%	5.4%	14.8%	17.3%	19.7%

White blood cells from cattle blood show a time dependent uptake of DK20 nanosponges. The entire WBC (white blood cells) population was subclassified into granulocytes (neutrophils) and other leukocytes such as lymphocytes and monocytes. Here, the other leukocyte group loaded twice as well (>50%) compared to the granulocyte group (∼25%). The loading was observed over a timeframe of 6 hours with the maximum loading completed after 3 hours.

**Fig. 9:**
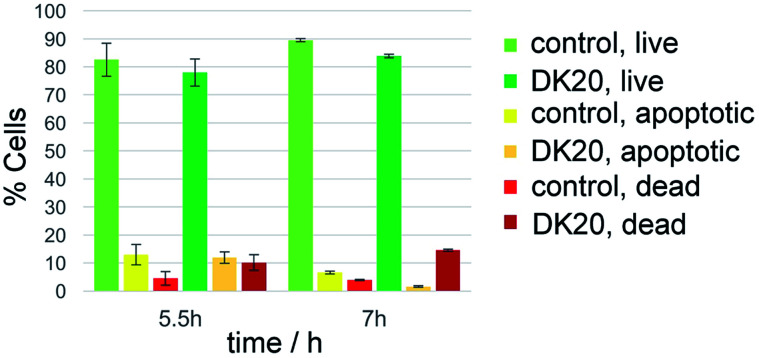
Survival of WBC when exposed to DK20 peptide nanosponges, compared to the survival of an unexposed WBC control group. No significant difference (*p* > 0.05) between the treated and untreated live cell populations was detected.

Survival of the WBC population was analyzed by detecting apoptotic cells with the annexin V fluorescent marker and dead cells with propidium iodine. The relative survival was measured after 5.5 and 7 hours of incubation with DK20 nanosponges and compared to a control group. The live cell population remained between 78% and 89% relative to the total cell count and with no significant difference between the control and the DK20 group for the duration of the experiment. Apoptotic cells were at approximately 12% after 5.5 hours, again with no significant difference between the two groups. The apoptotic cell count in the DK20 nanosponge group drops to 2% after seven hours while the dead cell count is significantly increased and measured at 15%. Our hypothesis is that this is observed due to the stress exerted on the WBS from endocytosis and processing of the DK20 nanosponges (Fig. 9).

## Discussion

Three main obstacles to efficient cell-mediated therapy of cancer and infectious diseases remain today: (1) fast uptake of drug formulations by the transport cells. (2) Effective migration of the transport cells to their intended target. (3) Efficient drug release by the transport cells once the target is reached. The nanosponges that are discussed here will be able to efficiently target neural progenitor cells^38,53^ and leucocytes, either *ex vivo* or, preferentially, in peripheral blood to utilize the advantages of autologous cells for patient-specific cell therapies. Because of their fast uptake kinetics, and virtually non-existent toxicity, the nanosponges are well-suited for loading numerous drug formulations into various types of transport cells. Furthermore, because of their low toxicity to the transport cells, they make a very important contribution to facilitating effective cell migration to targets *in vivo*, because the viability of the transport cells will remain high during the migration phase of several days. In our previous work, we have observed by means of fluorescence microscopy that type DK20 nanosponges retained their structures within the cytoplasm of monocyte/macrophage-like cells (RAW 264.7) for up to 72 h (ref. 38). Since RAW 264.7 cells belong to the group of leukocytes, this finding has importance for the study reported here. Finally, as we have demonstrated here by utilizing 5(6)-carboxyfluorescein as model drug, caspase-mediated drug release can be achieved. The observed increase in carboxyfluorescein emission was 38.2 ± 0.9% after 55 min in the presence of 1.0 × 10^−6^ M of caspase-6 in PBS, compared to PBS alone. Furthermore, the “caspase storm” during apoptosis has the potential of further degrading the nanosponges and to create numerous apoptotic bodies, to which the nanosponge-derived components will be adsorbed. Therefore, we anticipate a very high degree of drug release and consequent re-uptake *in vivo*.

We have utilized caspase-6 (MEROPS, ID: C14.005) in our studies, because it is one of the “effector caspases of apoptosis”.^54^ Caspases 3 and 6 are responsible for significant morphological changes in the nucleus at the onset of apoptosis. Caspase-6 cleaves nuclear lamina and the protein NuMa of the nuclear mitotic apparatus. Caspase-6 is also a suitable protease to cleave the DEVDGC motif of the nanosponges. According to this mechanism, drug release from the nanosponges within the transport cells can be triggered by apoptosis.^55^ It is noteworthy that the consensus sequence DEVGDC is also capable of reacting with the other effector caspases-2, -3, and -7.^39^

Neutrophils make up a significant percentage of leucocytes.^56^ As already discussed, neutrophils undergo apoptosis within hours after reaching tumors and metastases.^56^ This makes them very attractive autologous cells for tumor targeting. Neural progenitor cells constitute a second class of delivery cells, which can migrate to solid tumors and metastases in large numbers.^29,34,46^ However, the release of the payload has to be triggered by introducing apoptosis, as described above of by designing a TetOn gene regulation system, which silences a specific gene unless a tetracycline, such as doxycycline, is present.^10,57^

## Conclusion

The structure predictions for the supramolecular binary nanosponges (type DK20) through explicit solvent and then coarse-grained molecular dynamics (MD) simulations, have been confirmed by transmission electron microscopy and dynamic light scattering studies. The structural and dynamic understanding of the nanosponges has enabled several applications of these novel materials, which, principally, prove them as advanced biomaterials in cell-mediated drug transport to solid tumors/metastases and infectious diseases: caspase-activated (model) drug release was demonstrated with 5(6)-carboxyfluorescein-loaded nanosponges. PKH26-loaded nanosponges were essentially non-toxic to cultured neural progenitor cells (NPC).^38^ Targeting of leucocytes (WBC) in peripheral blood was successful. After 3 h of incubation, maximal uptake into non-granulocytic leukocytes and granulocytes in peripheral blood was observed. No significant difference between the untreated and DK20-nanosponge-treated live cell populations was detected. These results indicate that leucocytes in peripheral blood can be targeted by DK20 nanosponges without the requirement of previous isolation. We regard this as an important step towards cell-mediated therapy of tumors utilizing autologous cells as delivery vectors.

## Author contributions

The manuscript was written through contributions of all authors./All authors have given approval to the final version of the manuscript.

## Conflicts of interest

There are no conflicts to declare.

## Abbreviations

PBS: Phosphate-buffered saline buffer
AFM: Atomic force microscopy
TEM: Transmission electron microscopy
HBTU: (2-(1*H*-Benzotriazol-1-yl)-1,1,3,3-tetramethyluronium hexafluorophosphate)
DIEA: *N*,*N*-Diisopropylethylamine
CDI: (Carbonyl-di-imidazole)
TIPS: Triisopropylsilane
HEPES: 4-(2-Hydroxyethyl)-1-piperazineethanesulfonic acid

## Supplementary Material

RA-008-C8RA00717A-s001
