# Supplementary material for: Peptide nanosponges designed for rapid uptake by leukocytes and neural stem cells
Source: RSC Adv. 2018 Apr 30;8(29):16052–60. doi: 10.1039/c8ra00717a (PMC9080234; doi:10.1039/c8ra00717a)
Supplement: RA-008-C8RA00717A-s001 [file RA-008-C8RA00717A-s001.pdf]

Electronic Supplementary Material (ESI) for RSC Advances.
This journal is © The Royal Society of Chemistry 2018

Peptide Nanosponges Designed for Rapid Uptake by Leukocytes and Neural Stem Cells

Asanka S. Yapa[a‡], Hongwang Wang[a‡], Sebastian O. Wendel[bc‡], Tej. B. Shrestha[b‡], Nilusha L. Kariyawasam[a], Madumali Kalubowilage[a], Ayomi S. Perera[a†], Marla Pyle[b], Matthew T. Basel[b], Aruni P. Malalasekera[a†], Harshi Manawadu[a†], Jing Yu[a], Yubisela Toledo[a], Raquel Ortega[a], Prem S. Thapa[c], Paul E. Smith[a], Deryl L. Troyer[b*], Stefan H. Bossmann[a*]

[a] Department of Chemistry, Kansas State University, Manhattan, KS, USA

[b] Department of Anatomy & Physiology, Kansas State University, Manhattan, KS, USA

[c] Microscopy and Analytical Imaging Laboratory, University of Kansas, Lawrence, KS, USA

‡These authors have contributed equally

†Present addresses are provided in the main manuscript

[a] Department of Chemistry, Kansas State University, Manhattan, KS, USA

[b] Department of Anatomy & Physiology, Kansas State University, Manhattan, KS, USA

[c] Microscopy and Analytical Imaging Laboratory, University of Kansas, Lawrence, KS, USA

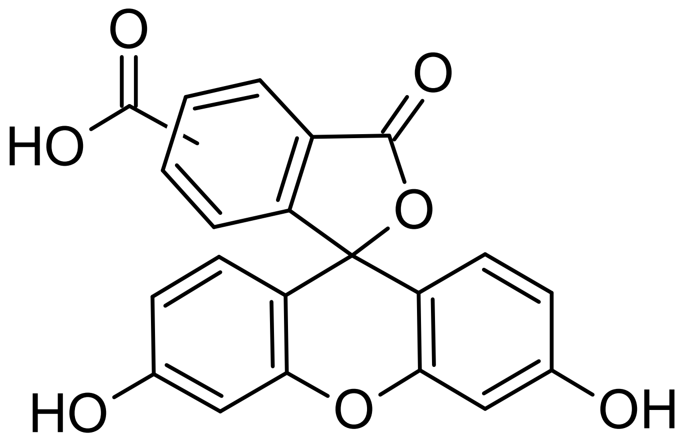

Figure S.1 5(6)-Carboxyfluorescein

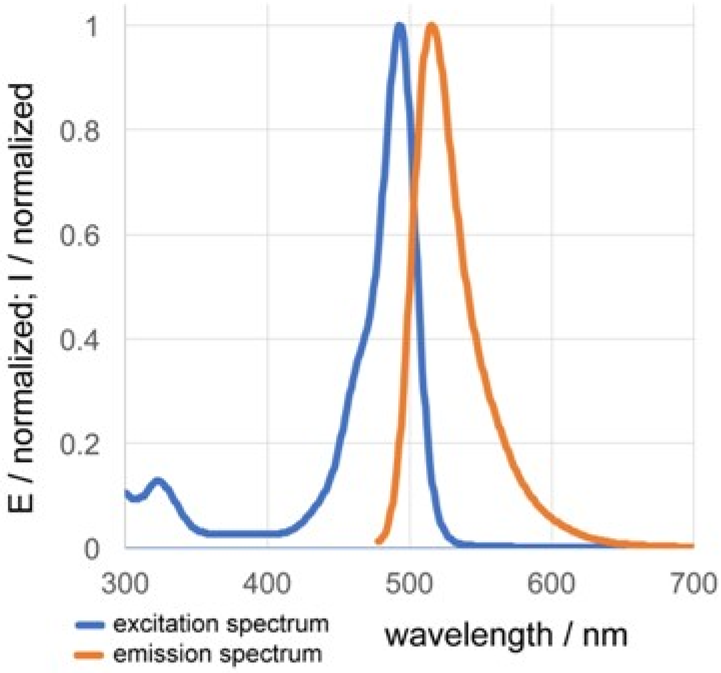

Figure S.2 Excitation and emission spectrum of 5(6)-carboxyfluorescein in PBS (pH = 7.4).

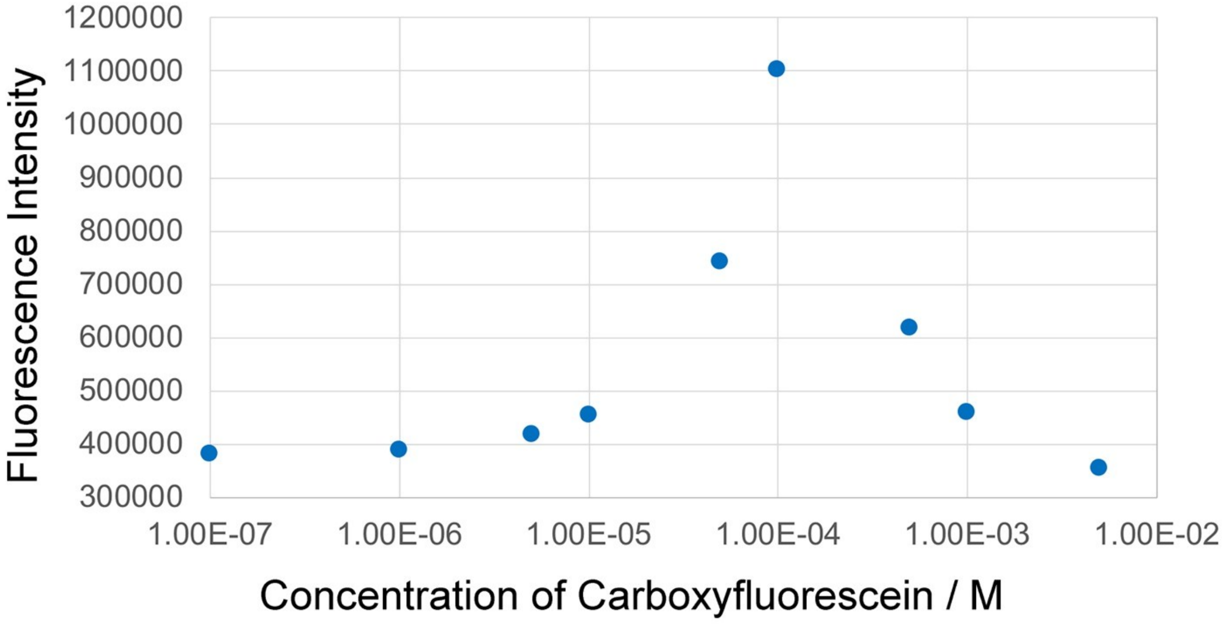

Figure S.3 Concentration dependence of the emission of 5(6)-carboxyfluorescein fluorescence in PBS (pH = 7.4), measuring using a Fluoromax-2 spectrometer, optical path length = 1.0 cm. λ_{EX} = 482 ± 1 nm, λ_{EM} = 515 ± 5 nm. In the concentration interval of 1.0 x 10-7 M and 5 x 10-5 M, the emission intensity of 5(6)-carboxyfluorescein is proportional to its concentration. Beyond this threshold, self-quenching of 5(6)-carboxyfluorescein is observed.

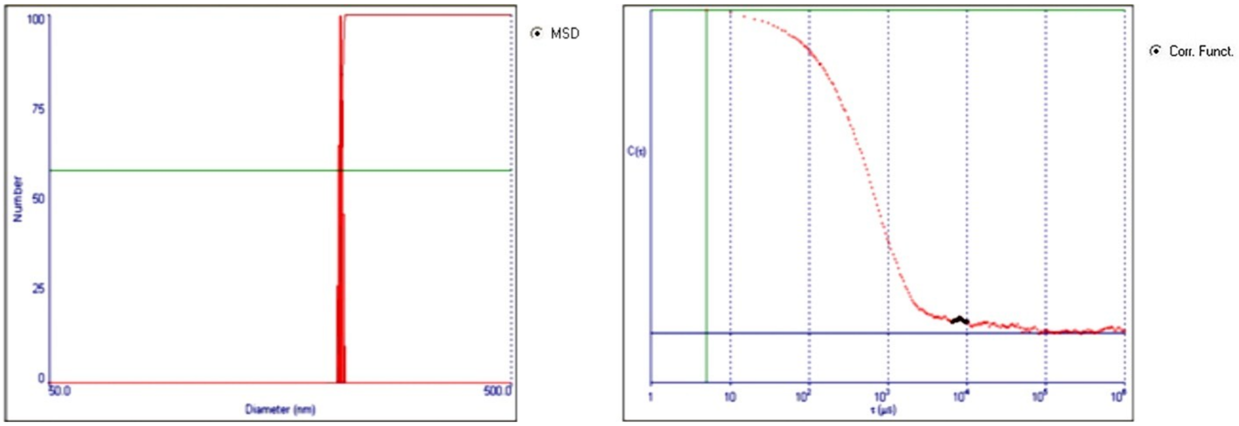

Figure S.4 DLS of 5(6)-Carboxyfluorescein-loaded type DK 20 nanosponges in PBS (phosphate-buffered saline, pH=7.4). The average diameter of the peptide nanosponges formed is 213 ± 25 nm. The nanosponges remained stable during 24h.

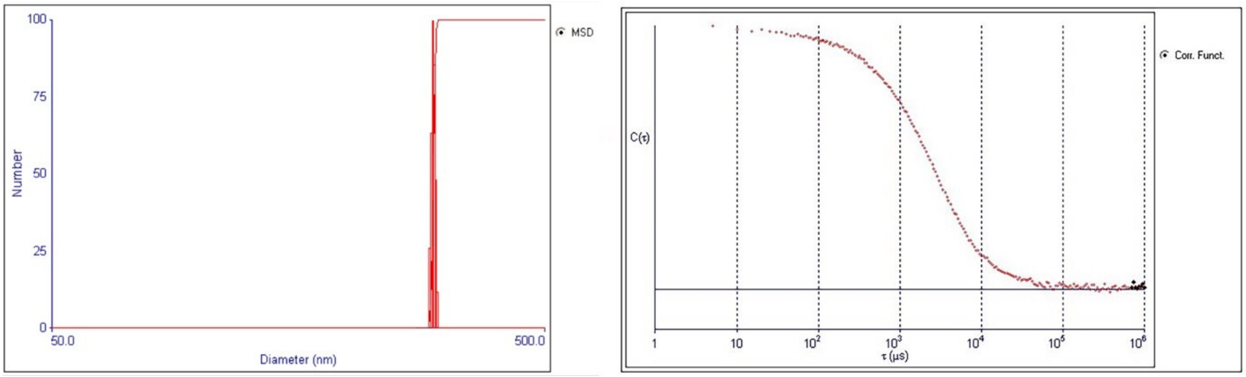

Figure S.5 DLS of 5(6)-Carboxyfluorescein-loaded type DK 20 nanosponges in PBS containing 5 percent of human serum from healthy volunteers, obtained from the Biorepository of the University of Kansas Cancer Center. The average diameter of the peptide nanosponges formed is 618 ± 25 nm. The nanosponges remained stable during 24h, indicating virtually no proteolytic cleavage.

Estimation of the Average Number of Carboxyfluorescein molecules per DK20 Nanosponge

In our earlier research, we have reported the molecular weight of type K20 nanosponges (11334.74 g mol^{-1}) and type D20 nanosponges (10439.51 g mol^{-1}). The concentration of DK20 nanosponges (1:1 mixture) in the described encapsulation experiments was 0.0005M for each component. The concentration of carboxyfluorescein was 10 micromolar (65 percent loading efficiency). The average size of the carboxyfluorescein-loaded nanosponge was 45 ± 10 nm (see Figure 6), corresponding to an average volume of each nanosponge of 4.77 x 10^{-23} m^3. Assuming that approx. 50% of the mass of the nanosponges is contributed by water and the approx. density of the nanosponges is close to 1.0 g cm^{-3}, we estimate the number of nanosponges per liter to 4.6 x 10^{17} at the reported concentration. The microheterogeneous system contains 3.91 x 10^{18} molecules L^{-1} of carboxyfluorescein. Based on these assumptions, the average number of carboxyfluorescein molecules per DK20 nanosponge is 8.5, or rather, between 8 and 9.
